# Dr. Alexander B. Mott

**Published:** 1889-09

**Authors:** 


DR. ALEXANDER B. MOTT died at his home in New York, August 12, 1889, after a brief sickness. The son of the great Valentine Mott, he was himself a surgeon of some renown. He was a surgeon of United States Volunteers during the late war, and a member of the Military Order of the Loyal Legion. He established the Mott Memorial Library, and taught surgery in Bellevue Hospital Medical College for some years.

